# Textural features of cervical cancers on FDG-PET/CT associate with survival and local relapse in patients treated with definitive chemoradiotherapy

**DOI:** 10.1038/s41598-018-30336-6

**Published:** 2018-08-08

**Authors:** Shang-Wen Chen, Wei-Chih Shen, Te-Chun Hsieh, Ji-An Liang, Yao-Ching Hung, Lian-Shung Yeh, Wei-Chun Chang, Wu-Chou Lin, Kuo-Yang Yen, Chia-Hung Kao

**Affiliations:** 1 Department of Radiation Oncology, China Medical University Hospital, Taichung, Taiwan; 2 School of Medicine, College of Medicine, China Medical University, Taichung, Taiwan; 3 Department of Radiology, School of Medicine, College of Medicine, Taipei Medical University, Taipei, Taiwan; 4 Department of Medical Research, China Medical University Hospital, China Medical University, Taichung, Taiwan; 5 Department of Computer Science and Information Engineering, Asia University, Taichung, Taiwan; 6 Department of Nuclear Medicine and PET Center, China Medical University Hospital, Taichung, Taiwan; 7 Department of Biomedical Imaging and Radiological Science, China Medical University, Taichung, Taiwan; 8 Graduate Institute of Biomedical Sciences, School of Medicine, College of Medicine, China Medical University, Taichung, Taiwan; 9 Department of Obstetrics and Gynecology, China Medical University Hospital, Taichung, Taiwan; 10 Department of Bioinformatics and Medical Engineering, Asia University, Taichung, Taiwan

## Abstract

We retrospectively reviewed the records of 142 patients with stage IB–IIIB cervical cancer who underwent ^18^F-FDG-PET/CT before external beam radiotherapy plus intracavitary brachytherapy and concurrent chemotherapy. The patients were divided into training and validation cohorts to confirm the reliability of predictors for recurrence. Kaplan–Meier analysis was performed and a Cox regression model was used to examine the effects of variables on overall survival (OS), progression-free survival (PFS), distant metastasis-free survival (DMFS), and pelvic relapse-free survival (PRFS). High gray-level run emphasis (HGRE) derived from gray-level run-length matrix most accurately and consistently predicted the presence of pelvic residual or recurrent tumors for both cohorts. In multivariate analysis, stages IIIA–IIIB (*P* = 0.001, hazard ratio [HR] = 4.07) and a low HGRE (*P* < 0.0001, HR = 4.34) were prognostic factors for low OS, whereas a low HGRE (*P* = 0.001, HR = 2.86) and nonsquamous cell histology (*P* = 0.003, HR = 2.76) were prognostic factors for inferior PFS. The nonsquamous cell histology (*P* < 0.0001, HR = 9.19) and a low HGRE (*P* = 0.001, HR = 4.69) were predictors for low PRFS. In cervical cancer patients receiving definitive chemoradiotherapy, pretreatment textural features on ^18^F-FDG-PET/CT can supplement the prognostic information.

## Introduction

Chemoradiotherapy (CRT) has been the standard of care for patients with locally advanced cervical cancer worldwide for more than a decade. However, the outcomes of these patients have not been ideal in terms of local control or survival. Several pretreatment clinical characteristics have been used to predict the risk of local recurrence or distant metastasis^1,2^. At present, ^18^F-fluorodeoxyglucose (^18^F-FDG) positron emission tomography (PET)–computed tomography (CT) is widely used for staging and monitoring treatment outcomes in various cancers including cervical cancer^3,4^. Tumors often present with biological heterogeneity corresponding to hypoxia, fibrosis, angiogenesis, or high cellular proliferation^5^, and such biological characteristics are of interest because they are often associated with aggressiveness or sensitivity to a specific therapy^6^. Therefore, understanding the prognostic role of textural features is imperative. The measurement of intratumoral metabolic heterogeneity by using textural indices on ^18^F-FDG PET-CT has recently been proposed as an adjunct to predict a tumor’s response to a therapy in various cancers^7–15^. Compared with a single standard uptake value (SUV), metabolic tumor volume (MTV) or total lesion glycolysis (TLG), textural features, which indicate the uptake distribution within a tumor, can theoretically provide more insights into the underlying biology.

Identifying patients predisposed to tumor recurrence is crucial for counseling them on treatment options and prognosis and for making treatment decisions. Although CRT is a mainstay for advanced cervical cancer, pertinent studies investigating the association of textural features on ^18^F-FDG PET-CT with CRT outcomes are limited^11–14^. We hypothesized that the pretreatment textural indices of cervical cancer on ^18^F-FDG PET-CT can be used along with clinical parameters to predict CRT-based treatment outcomes. Comprehensive textural indices were investigated to understand their predictive role^16^.

## Materials and Methods

### Study population

In this retrospective cohort study, we included 142 patients newly diagnosed as having cervical cancer at China Medical University Hospital between July 2009 and December 2015. All patients had undergone ^18^F-FDG PET-CT for pretreatment staging and received allocated external beam radiotherapy and intracavitary brachytherapy. Concurrent chemotherapy consisted of cisplatin administered weekly at a dose of 40 mg/m^2^. All patients had normal serum glucose levels before undergoing PET-CT. The eligibility criterion with respect to the minimum size for primary tumors was a maximal diameter of ≥2 cm on a CT. Performing textural analysis of small lesions such as lymph node is difficult because of the low number of voxels involved^17^. Thus, we considered only primary tumors for textural analysis. This study was approved by a local institutional review board [DMR99-IRB-010(CR6)]. We performed tumor staging according to the International Federation of Gynecology and Obstetrics (FIGO) staging system and observed that 37, 78, and 27 patients had stage I, II, and III cervical cancer, respectively. The median age of patients was 55 years. The histological type was squamous cell carcinoma in 114 patients and nonsquamous cell carcinoma in 28 patients. Because PET-CT has high sensitivity and specificity in detecting the nodal status in cervical cancer^18,19^, the diagnosis of pelvic lymph node (PLN) was based on PET-CT^17^. Patients with paraaortic lymph node metastasis on PET-CT were excluded. The characteristics of the patients are listed in Table 1.

**Table 1:** Patients’ characteristics (N = 142).

Variables	Value
Age (year)	median 55 (range, 28~81)
FIGO stage	
IB	37 (26%)
IIA-IIB	78 (55%)
IIIA-IIIB	27 (19%)
Histology	
squamous cell carcinoma	114 (80%)
adenocarcinoma	28 (20%)
Pelvic LN metastasis	
negative	81 (57%)
positive	61 (43%)
SUV_max_	mean 11.3 ± 6.0 (range, 2.9~37.0)
MTV (mL)	mean 28.1 ± 42.7 (range, 2.5~450.0)
TLG_mean_ (g)	mean 216.1 ± 280.5 (range, 4.7~1800.9)
External beam radiotherapy (cGy)	
whole pelvis (Gy)	median 45 (range, 39.6~54)
bilateral parametrium boost with central shielding (Gy)	median 54 (range, 50.4~57.6)
pelvic lymph node boost (Gy)	median 60 (range, 54~66)
Brachytherapy	
2 dimensional brachytherapy	65
cumulative EQD2 of point A (Gy_10_)	77.3 ± 7.3
6 Gy/point A	n = 49
5 Gy/point A	n = 16
3 dimensional brachytherapy	77
cumulative EQD2 of D90 of HR-CTV (Gy_10_)	87.1 ± 9.2

Abbreviations: FIGO = International Federation of Gynecology and Obstetrics; MTV = metabolic tumor volume using fixed thresholds of 50% of the SUV_max_; TLG_mean_ = mean SUV multiplied by volume using fixed thresholds of 50% of the SUV_max_; EQD2 = equivalent dose in 2 Gy; and HR-CTV = high-risk clinical target volume.

### Training and validation cohorts

The 142 patients were divided into two cohorts (77 for training, 65 for validation) to confirm the reliability of predictors for pelvic recurrence or disease progression. The first cohort came from patient lists between July 2009 and June 2013, whereas the validation cohort consisted of patients with pretreatment PET-CT after June 2013. Basically, the two cohorts received the same protocol of PET-CT imaging and interpretation which would be mentioned below.

### PET-CT imaging

All patients were scanned using a PET-CT scanner (PET/CT-16 slice; Discovery STE; GE Medical System, Milwaukee, WI, USA). Patients were requested to fast for at least 4 hours before undergoing ^18^F-FDG PET-CT, and the technique was conducted approximately 60 min after the administration of 370 MBq of ^18^F-FDG regardless of body weight. Thus, ^18^F-FDG uptake was determined by calculating the SUV. The procedure was described previously^20^.

### Definition of SUV_max_, MTV, and TLG

The SUV of the highest local maximum within the spatial extent of a cervical tumor is defined as the SUV_max_. In PET images, all voxels with SUVs higher than SUV_max_ × 0.5 are grouped into connected components by using 18-connected neighbors. The connected component containing the SUV_max_ of a cervical tumor is defined as the MTV. If it connects to the nonpathological bladder, the MTV is partitioned into several regions through the watershed transform^21^. Regions belonging to the bladder are manually excluded from partition results to acquire the MTV of a cervical tumor. For this study, several methods including a fixed threshold at 40% and 50% of SUV_max_, adaptive threshold, gradient-based method, and region growing have been examined^12,22–26^. The MTVs connecting with the bladder was a major problem for most segmentation methods. Although it is undetermined to limit the inclusion of adjacent normal organ in the heterogeneity analysis, the decision of the spatial extent of a MTV delineated by a fixed threshold at 50% of SUV_max_ was trade-offs between the adequate separation and the sufficient intratumoral uptakes. The TLG_mean_ was calculated by multiplying the SUV_mean_ of the cervical tumor by the MTV.

### Calculation of textural indices

Discretization of the SUVs within the previously delineated MTV was the fundamental for the calculation of textural indices. Two types of the discretization method were employed to divide the SUV range within the MTV into a fixed number of bins or into a fixed bin size. The discretization method using a fixed number of bins divided the SUV range into 4, 8, 16, 32, 48, 64, 80, 96, 112 or 128 bins, respectively. The SUV range was resampled by a width of 0.01, 0.025, 0.05, 0.075, 0.1, 0.25, 0.5, 0.75, or 1 g/ml when the discretization method using a fixed bin size. We calculated four textural matrices for evaluating heterogeneity indices, namely the gray-level co-occurrence matrix (GLCM)^27^, neighboring gray-level dependence matrix (NGLDM)^28^, gray-level run-length matrix (GLRLM)^29^, and gray-level size zone matrix (GLSZM)^30^. Because the measurements of the GLCM and GLRLM are directional, the neighbor of each voxel (x, y, z) within a cervical tumor was defined as that located with the offset (0, 1, 0). A total of 31 indices were derived from the four matrices, as listed in Appendix 1.

### Treatment

All patients underwent CT-based planning with custom immobilization. An intensity-modulated radiotherapy plan consisted of seven coplanar fields using 10-MV photons. The prescription dose to the whole pelvis was 45 Gy in 25 fractions over 5 weeks.

As previously described^31^, the clinical target volume (CTV) included the gross disease, cervix, parametrium, uterus, superior half of the vagina, cardinal ligament, presacral region, and regional lymph nodes (common, internal, and external iliac). The planning target volume (PTV) was extended from the CTV to account for organ motion and setup uncertainty. We applied a 15-mm planning margin around the cervix, a 10-mm margin around the uterus and vagina, and an 8-mm margin around the remainder of the CTV.

Target planning constraints were standardized as follows: (1) >97% of the PTV received >97% of the prescription dose, (2) <1% of the PTV received <93% of the prescription dose, and (3) <5% of the PTV received >107% of the prescription dose. When prescribing a dose of 45 Gy to the whole pelvis, the following normal tissue planning constraints were consistently applied: (1) <50% of the volume of the rectum received >45 Gy, (2) <50% of the volume of the bladder received >45 Gy, (3) <10% of the volume of the nonrectal bowel received >45 Gy, (4) the spinal cord received the maximum dose of <40 Gy, and (5) the kidney dose was <35% at 16 Gy. No special constraint was used for the adjacent bone or bone marrow.

Following pelvic irradiation, the bilateral parametrium was boosted from 50.4 to 54 Gy through the anterior and posterior parallel field technique by using rectangular central shielding with a width of 4 cm. Thereafter, the involved PLNs were sequentially boosted with an external beam dose of up to 60 Gy for PLNs with the maximal diameter of <2 cm and 64–66 Gy for those with maximal diameters of ≥2 cm.

### Brachytherapy

After adequate tumor regression, high-dose rate intracavitary brachytherapy was performed using an Ir-192 remote afterloading technique, one or two times per week, concurrently with pelvic irradiation or parametrial boosting^32^. Before January 2013, the standard prescribed dose for each brachytherapy was 6.0 Gy to Point A for four sessions for squamous cell carcinoma, and five sessions for adenocarcinoma or adenosquamous cell carcinoma. The Point A dose was reduced to 5.0 Gy for those with higher reference doses to the rectum or bladder or those aged >70 years. In this two dimensional (2D) brachytherapy period, the total prescribed Point A doses (external beam radiotherapy + brachytherapy) of a radiobiological equivalent dose in 2-Gy fractions (EQD2) ranged from 69.3 to 84.3 (median, 77.3) Gy_10_. After January 2013, 77 patients were treated with three dimensional (3D) image-based brachytherapy planning according to the Groupe Européen de Curiethérapie recommendations and the European Society for Radiotherapy and Oncology guidelines^33^. The mean high-risk CTV (HR-CTV) was 32.8 ± 17.5 mL, whereas the mean cumulative EQD2 of D90 of HR-CTV (Gy_10_) was 87.1 ± 9.2 Gy.

### Chemotherapy

Chemotherapy consisted of cisplatin administered weekly at a dose of 40 mg/m^2^ intravenously, for a maximal dose of 60 mg. The detailed drug administration protocol has been described in our previous study^31^. Chemotherapy was withheld if we observed any hematological toxicity of more than or equal to grade 3.

### Follow-up

The follow-up protocol was consistent between the two different treatment periods. After the completion of radiotherapy, patients were regularly followed up every 1 to 2 months for the first year, and every 3 months thereafter. A pelvic examination was performed during each follow-up. In addition, the levels of serum tumor markers, namely squamous cell carcinoma antigen (SCC-Ag) and carcinoembryonic antigen (CEA), were examined. A radiographic examination was performed every 3 to 6 months. In patients with evidence of central pelvic recurrence, salvage hysterectomy or pelvic exenteration was performed, if feasible. In patients with distant metastasis, systemic chemotherapy was performed depending on their tolerability.

### Statistical analysis

This study was conducted to investigate textural indices on ^18^F-FDG PET-CT to determine the optimal approach for predicting CRT-based outcomes. To examine correlations between the aforementioned parameters and tumor recurrence, receiver operating characteristic (ROC) curves were constructed to evaluate the optimal predictive performance among the various textural indices, conventional PET-CT parameters, and clinical parameters, such as the stage and presence of PLN metastasis. In addition, binary logistic regression analysis was performed to determine the independent factors among all the features for predicting clinical outcome. The correlation between textural features and clinical parameters was examined using Pearson’s correlation coefficient. The outcome endpoints were overall survival (OS), progression-free survival (PFS), distant metastasis-free survival (DMFS), and central pelvic relapse-free survival (PRFS), all of which were calculated using the Kaplan–Meier method. The log-rank test and Cox regression analysis were performed to examine the effects of explanatory variables on these endpoints. The stage, age, nodal status, histology, serum tumor markers, conventional PET-parameters, and textural indices with a significant value in ROC analysis were included for multivariate analysis. Patient survival was measured from the date of initiation of radiotherapy to the last follow-up. Two-tailed tests were used, and *P* < 0.05 was considered statistically significant. All calculations were performed using SPSS, Version 13.0 for Windows (SPSS Inc., Chicago, IL, USA).

### Ethical approval

This study was approved by a local institutional review board (CMUH103-REC2-093FR).

### Informed consent

The institutional review board specifically waived the consent requirement.

## Results

### Treatment outcomes

Based on pretreatment PET-CT, we identified ≥1 PLN metastasis in 61 patients (Table 1). After a median follow-up duration of 40 (range, 7–84) months, 115 patients were alive and 27 patients had died. Moreover, 101 patients had no evidence of disease progression, whereas 41 patients had disease progression (infield recurrence, distant metastasis, and both in 22, 27, and 9 patients, respectively). Among the 22 patients with infield recurrence, none experienced sole PLN relapse. In summary, 120 patients remained relapse free at primary sites, whereas 22 patients experienced central pelvic recurrence. The presence of pelvic recurrence and distant metastasis were identified in 12 and 16 patients for the training cohort, whereas 10 and 11 for the validation cohorts, respectively.

### Selection of the discretization method and parameter setting

The selection of the discretization method and its parameter setting was determined by the performance of the indices in predicting pelvic recurrence. In the process of the discretization, the SUV range within the MTV was quantized by a specific parameter and then used to derive textural features. Then, the advantage of a discretization method was evaluated by the performances of the derived textural indices by the ROC curves in terms of pelvic recurrence. For the textural indices obtained from all parameters per discretization method, all acquired areas were listed in Appendix 2.

### Comparison of the predictive ability of different parameters and textural indices for local failure and distant metastasis for training and validation cohorts

The four groups of textural indices and the conventional PET-CT parameters were retrieved. The final selection of the quantization method is determined by the performance in predicting disease recurrence. The optimal predictive values were achieved when SUVs within the MTV of a cervical tumor were quantized into four equal bins. As shown in Table 2, when the area under the ROC curve was reported if the value was greater than 0.6 or less than 0.4 in the training cohort, several textural features among the GLCM, GLRLM, and GLSZM met the criteria. Of them, high gray-level run emphasis (HGRE) derived from GLRLM most accurately and consistently predicted the presence of central residual or recurrent tumors for both cohorts (AUC: 0.30 and 0.34 for the training and validation cohorts, respectively). In addition, the logistic regression analysis showed HGRE had the highest predictive score in both cohorts. Owing to limited events in both cohorts; however, this feature failed to reach a statistical significance (*P* = 0.056 and *P* = 0.08 for the training and validation cohorts, respectively). The values of HGRE demonstrated an inverse correlation with pelvic recurrence. When taken whole population together, the AUC for HGRE was 0.31 ± 0.06, *P* = 0.006, whereas the logistic regression analysis identified HGRE was the only textural feature in determining pelvic recurrence [Odds ratio (OR) 1.98, 95% confidence interval (CI) 1.16–3.39, *P* = 0.01]. Based on Youden’s index, we found an optimal cutoff for HGRE of 3.68 (AUC: 0.27 and 0.34 for the training and validation cohorts; 0.31 ± 0.06, *P* = 0.005 for whole population).

**Table 2:** Predictive textural indices for pelvic recurrence and the area under the ROC curve in training and validation cohorts (report textural features with AUC value ≧0.6 or ≦0.4 in training cohort)

Classification of matrix	Index	AUC/*P* value Training cohort	AUC/*P* value. Validation cohort
Tumor marker	SCC-Ag	0.31 ± 0.08/0.05	0.39 ± 0.10/0.25
	CEA	0.50 ± 0.09/0.98	0.66 ± 0.10/0.09
Conventional PET-related parameter	SUV_max_	0.57 ± 0.10/0.50	0.44 ± 0.08/0.23
	MTV	0.46 ± 0.09/0.71	0.52 ± 0.08/0.83
	TLG_mean_	0.49 ± 0.10/0.92	0.47 ± 0.08/0.76
Gray Level Cooccurrence Matrix (GLCM)	Energy	0.64 ± 0.08/0.14	0.58 ± 0.09/0.38
	Entropy	0.34 ± 0.07/0.10	0.42 ± 0.09/0.40
Gray-Level Run Length Matrix (GLRLM)	LGRE	0.70 ± 0.08/0.04	0.60 ± 0.10/0.29
	HGRE	0.30 ± 0.08/0.04	0.34 ± 0.09/0.08
	SRLGE	0.69 ± 0.07/0.06	0.65 ± 0.09/0.10
	SRHGE	0.35 ± 0.08/0.12	0.36 ± 0.08/0.13
	LRHGE	0.35 ± 0.09/0.14	0.36 ± 0.09/0.12
Neighborhood Gray-Level Different Matrix (NGLDM)	None		
Gray-Level Zone Length Matrix (GLSZM)	SZHGE	0.62 ± 0.09/0.23	0.64 ± 0.09/0.12
	LGZE	0.35 ± 0.09/0.17	0.44 ± 0.10/0.50
	HGZE	0.64 ± 0.09/0.12	0.45 ± 0.08/0.60

Abbreviations: AUC = the area under the ROC curve; SCC-Ag = squamous cell carcinoma antigen; CEA = carcinoembryonic antigen; LGRE = low gray-level run emphasis; HGRE = high gray-level run emphasis; SRLGE = short-run low gray-level emphasis; SRHGE = short-run high gray-level emphasis; LRHGE = long-run high gray-level emphasis; SZHGE = short-zone high gray-level emphasis; HGZE = high gray-level zone emphasis; LGZE = low gray-level zone emphasis.

None of the textural features appeared to be prognostic for distant failures for both cohorts or whole population. In addition, ROC curves showed that HGRE was a sole textural index that consistently predicted the presence of disease progression in both cohorts (Table 3). Using the logistic regression analysis, HGRE achieved the best predictive value for progressive diseases for the training or validation cohorts. Likewise, this index failed to attain a consistently statistical significance due to restricted events (*P* = 0.03 and *P* = 0.09 for the training and validation cohorts, respectively). When lumping two cohorts together, HGRE was the sole textural feature in predicting disease progression (OR 1.75, 95% CI 1.14–2.65, *P* = 0.008).

**Table 3:** Predictive textural indices for disease progression and the area under the ROC curve in training and validation cohorts (report textural features with AUC value ≧0.6 or ≦0.4 in training cohort).

Classification of matrix	Index	AUC/*P* value Training cohort	AUC/*P* value. Validation cohort
Tumor marker	SCC-Ag	0.43 ± 0.07/0.31	0.39 ± 0.09/0.16
	CEA	0.54 ± 0.07/0.58	0.54 ± 0.08/0.61
Conventional PET-related parameter	SUV_max_	0.53 ± 0.07/0.74	0.55 ± 0.08/0.54
	MTV	0.51 ± 0.07/0.93	0.54 ± 0.07/0.62
	TLG_mean_	0.50 ± 0.07/0.99	0.53 ± 0.07/0.68
Gray Level Cooccurrence Matrix (GLCM)	Entropy	0.39 ± 0.07/0.30	0.37 ± 0.07/0.09
Gray-Level Run Length Matrix (GLRLM)	LGRE	0.60 ± 0.07/0.16	0.62 ± 0.08/0.12
	HGRE	0.36 ± 0.07/0.05	0.33 ± 0.07/0.03
	SRHGE	0.37 ± 0.07/0.08	0.41 ± 0.07/0.25
Neighborhood Gray-Level Different Matrix (NGLDM)	None		
Gray-Level Zone Length Matrix (GLSZM)	None		

Abbreviations: as Table 2.

Among the four groups of indices, the optimal cutoff of HGRE was used in grouping the patients. The textural index using HGRE groups, conventional PET-related parameters, the median value of SCC-Ag (5 ng/mL), and the optimal value of CEA (10 ng/mL)^34^, combined with the stage, histology, and lymph node status, were selected for multivariate Cox regression model for survival analysis.

### Prognostic factors for OS, PFS, PRFS, and DMFS

**Figure 1:**
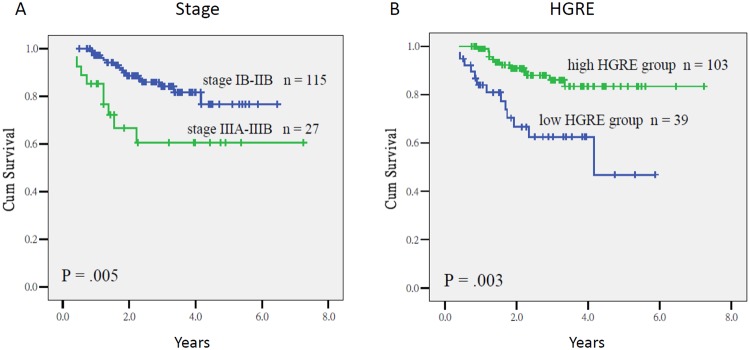
Overall survival in patients with stage IB–IIB and IIIA–IIIB cervical cancer (1A), and a high HGRE value (>3.68) and a low HGRE value (≤3.68) (1B) (*P* = 0.005 and *P* = 0.003, respectively).

**Figure 2:**
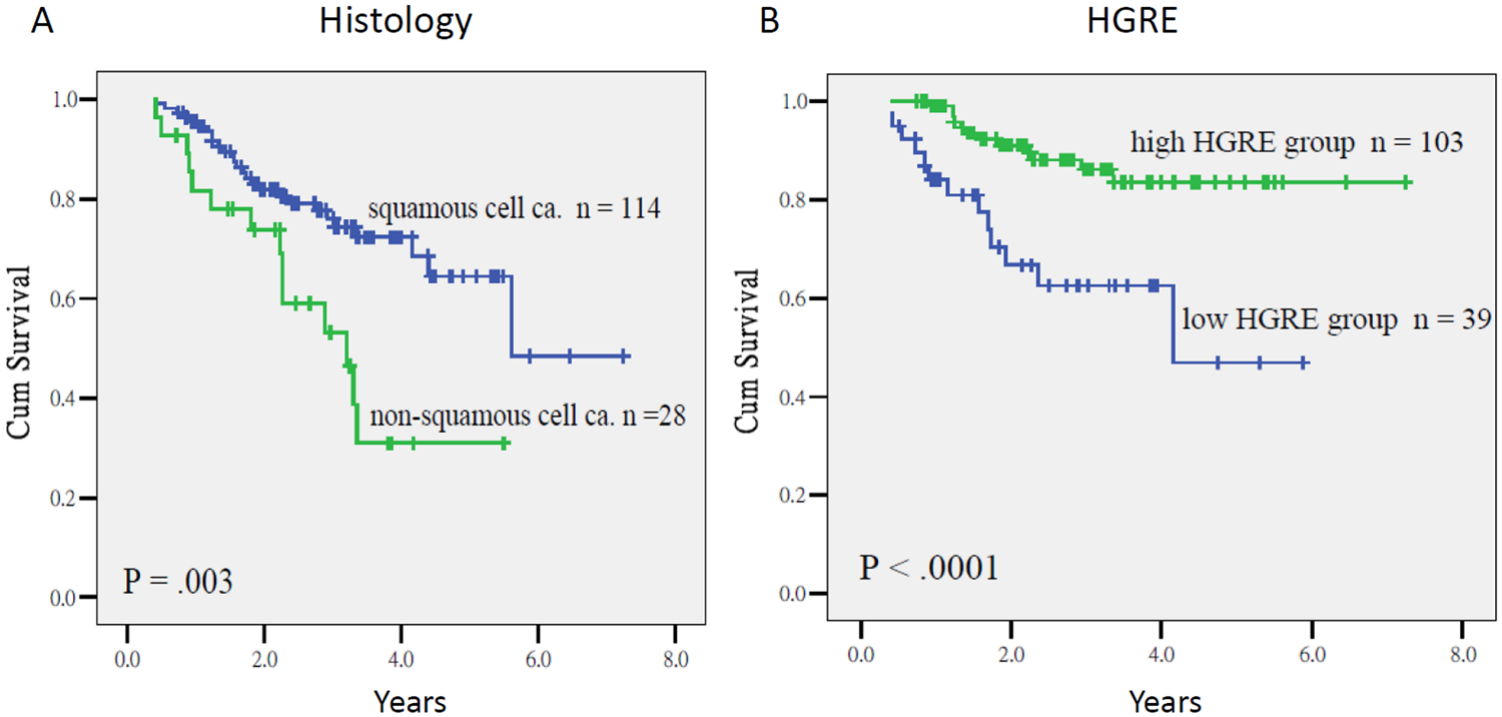
Progression-free survival in patients who had tumors with squamous cell carcinoma and nonsquamous cell histology (2A), and those who had an HGRE value > 3.68 and ≤3.68 (2B) (*P* = 0.003, and *P* < 0.0001, respectively).

Because of the limited events in both cohorts as mentioned above, the data of whole population were used for survival analyses. As summarized in Table 4, the Cox regression analysis indicated that the stage IIIA–IIIB [*P* = 0.001, Hazard ratio (HR) = 4.07, 95% CI = 1.72–9.61] and a low HGRE (*P* < 0.0001, HR = 4.34, CI = 1.96–10.00) were prognostic factors for low OS, whereas the nonsquamous cell histology (*P* = 0.003, HR = 2.76, CI = 1.43–5.33), and a low HGRE (*P* = 0.001, HR = 2.86, CI = 1.53–5.32) were prognostic factors for inferior PFS. The 3-year OS of the patients with stage IB–IIB and IIIA–IIIB diseases was 85% and 60%, respectively (*P* = 0.005), and among patients who had tumors with a high and low HGRE values, it was 86% and 62%, respectively (*P* = 0.003; Fig. 1). The 3-year PFS of the patients who had tumors with squamous cell carcinoma and nonsquamous cell histology was 75% and 53% (*P* = 0.003), respectively, and of those who had tumors with a higher and lower HGRE values was 88% and 62%, respectively (*P* < 0.0001; Fig. 2).

**Table 4:** Multivariate analyses using the Cox regression model for overall survival (OS), progression-free survival (PFS), and pelvic relapse-free survival (PRFS).

Variables	OS				PFS				PRFS			
	Univariate	Multivariate			Univariate	Multivariate			Univariate	Multivariate		
	*P*	*P*	HR	95% CI	*P*	*P*	HR	95% CI	*P*	*P*	HR	95% CI
Clinical parameters												
FIGO stage												
IIIA-IIIB vs. IB-IIB	0.005	0.001	4.07	1.72~9.61	0.31				0.94			
IIB-IIIB vs. IB	0.59				0.18				0.32			
N-stage												
PLN (+) vs. (−)	0.37				0.49				0.84			
Histotology												
non-squamous cell ca. vs.	0.79				0.003	0.003	2.76	1.43~5.33	<0.0001	<0.0001	9.19	3.41~24.82
squamous cell ca.												
SCC-Ag												
≧5 vs. <5 ng/dL	0.93				0.30				0.07			
CEA												
≧10 vs. <10 ng/dL	0.23				0.64				0.93			
Brachytherapy												
3D vs. 2D	0.11				0.21				0.59			
Conventional PET-CT parameters												
SUV_max_												
≧10.1 vs. <10.1	0.88				0.76				0.74			
MTV (mL)												
≧18.6 vs. <18.6	0.48				0.55				0.62			
TLG_mean_ (g)												
≧140.2 vs. <140.2	0.12				0.50				0.54			
Textural index												
HGRE												
low (≤3.68) vs. high (>3.68)	0.001	<0.0001	4.34	1.96~10.0	0.001	0.001	2.86	1.53~5.32	<0.0001	0.001	4.69	1.93~11.49

Abbreviations: HR = hazard ratio; FIGO = International Federation of Gynecology and Obstetrics; SCC-Ag = squamous cell carcinoma antigen; CEA = carcinoembryonic antigen, MTV = metabolic tumor volume using fixed thresholds of 50% of the SUV_max_; TLG_mean_ = mean SUV multiplied by volume using fixed thresholds of 50% of the SUV_max_; HGRE = high gray-level run emphasis.

**Figure 3:**
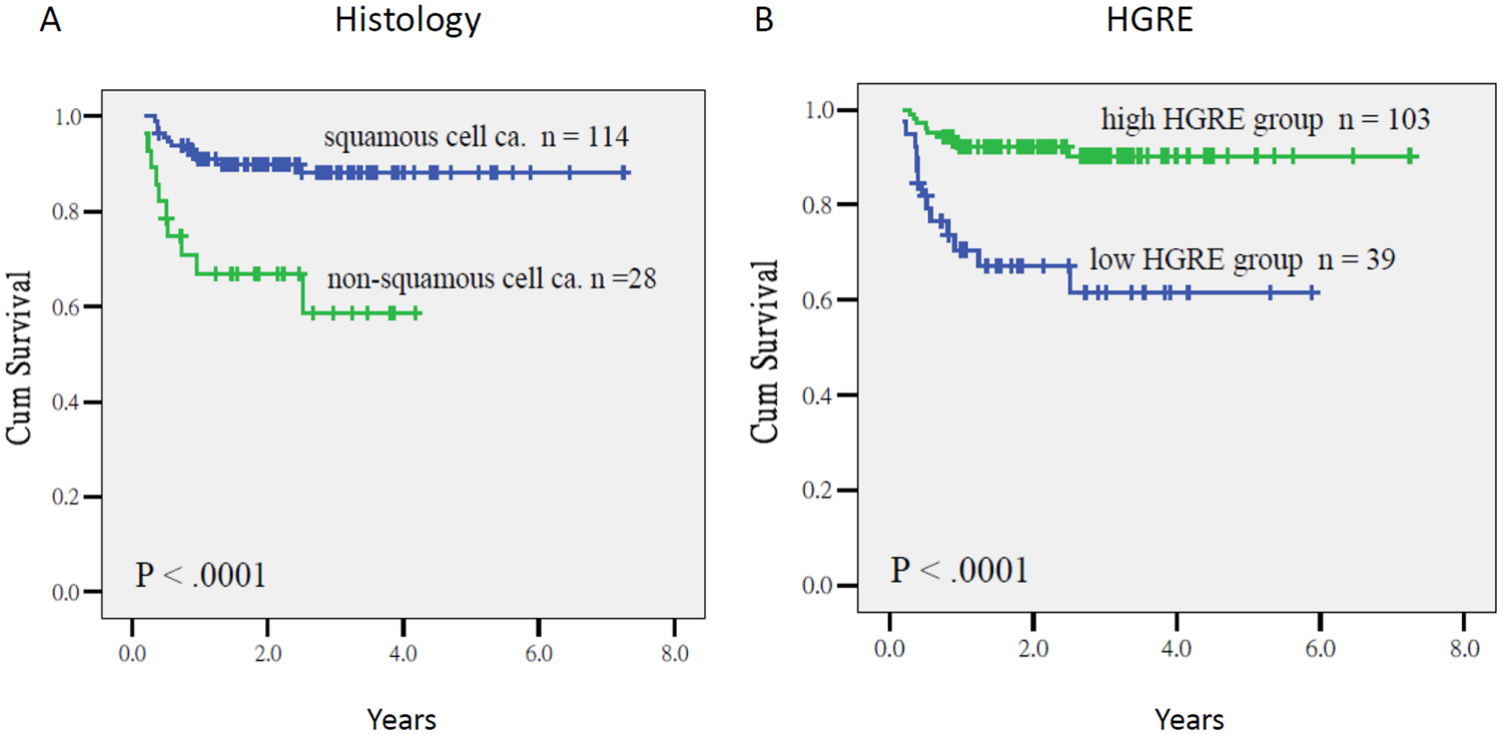
Pelvic relapse-free survival in patients who had tumors with the squamous cell carcinoma and nonsquamous cell histology (3A), and those with an HGRE value >3.68 and ≤3.68 (3B) (*P* < 0.0001, and *P* < 0.0001, respectively).

Tumors with nonsquamous cell histology (*P* < 0.0001, HR = 9.19, CI = 3.41–24.82) and a low HGRE (*P* = 0.001, HR = 4.69, CI = 1.93–11.49) were predictors of poor PRFS. The 3-year PRFS of the patients who had tumors with squamous cell carcinoma and nonsquamous cell histology was 89% and 58%, respectively (*P* < 0.0001), and that of the patients with a high and low HGRE value was 90% and 62%, respectively (*P* < 0.0001; Fig. 3). In addition, Pearson’s correlation coefficient analysis indicated no close relationship between the nonsquamous cell histology and HGRE (*P* = 0.75, R = −0.27).

None of the textural features or PET-CT related parameters showed a prognostic factor for DMFS, and the major determinant was the presence of PLN or the nonsquamous cell histology (*P* = 0.04, HR = 2.19, CI = 1.01–4.72 and *P* = 0.03, HR = 2.39, CI = 1.07–5.33, respectively).

In the multivariate analysis, age, tumor markers, conventional PET-CT parameters, or brachytherapy schemes (2D versus 3D) were not identified as independent prognostic factors for the aforementioned four endpoints.

### Subgroup analysis in patients with squamous cell carcinoma

To test the ability of the same textural features to supplement the prognostic information in patients with the squamous cell carcinoma histology (n = 114), a subgroup analysis was performed in this patient setting. The multivariate analysis indicated that a low HGRE value (*P* = 0.002, HR = 3.66, CI = 1.61–8.33) remained a prognostic factor for low OS, whereas the same value (*P* = 0.01, HR = 4.42, CI = 1.40–13.89) remained a prognostic factor for inferior PRFS. Tumors with a low HGRE value demonstrated a marginal impact on PFS in the univariate analysis. The 3-year OS of the patients who had tumors with a high and low HGRE was 84% and 59% (*P* = 0.001), respectively, whereas the 3-year PRFS of the patients with a high and low HGRE value was 91% and 74% (*P* = 0.006), respectively, as depicted in Appendix 3. The ROC curves indicated that none of the textural features appeared to be a prognostic factor for distant failures.

### Subgroup analysis in patients with FIGO stage IB-IIB diseases

As shown in Appendix 4, patients with stage IB-IIB (n = 115) were further analyzed to examine the ability of the same textural parameter to enhance the prognostic information already supplied by FIGO stage. The HGRE values indicated superior discrimination of OS and PRFS, respectively. This feature showed a similar trend of separating OS curves in patients with stage IIIA-IIIB tumor; however, there was no statistically significance due to a small sample size.

## Discussion

Recently, numerous studies have been published that investigate the clinical value of PET uptake heterogeneity in various tumor types, including esophageal, lung, rectal, breast, head and neck, and lymphoma^35^. In cervical cancer, an advanced FIGO stage and lymph node metastasis are two well-known prognostic factors; however, the role of textural features in providing a complementary or an additional value along with the stage or lymph nodes status has not been well established. In the field of exploiting intratumoral heterogeneity on ^18^F-FDG PET-CT, our study was the first to combine a comprehensive range of textural indices with clinical parameters to predict the outcome of CRT-based treatments in patients with cervical cancer. We originally suggest that tumor heterogeneity determined using a pretreatment textural index of HGRE can supplement the FIGO stage, histological type, or lymph nodes status in the field of precision medicine for cancer treatment, whereas the sole use of the conventional PET-related parameters failed to add further prognostic information (Tables 2, 3 and 4). In addition, our findings can be reproduced in patients with a histological type of squamous cell carcinoma. To verify the value of texture analysis, future studies should include multi-centric harmonized datasets with a stratification of the stage, histological type, and lymph node status. By using this method, a combination of textural indices would become more informative for a specific therapy.

To date, comparing the results from studies currently available and drawing solid conclusions with respect to the clinical value of textural analysis in PET imaging is challenging. This is due to large variability in the implemented methodologies associated with the workflow complexity^35^. To reduce disparities among studies and to avoid neglecting useful indices, we adopted the definitions of comprehensive matrix indices described by Orlhac *et al*.^16^. The bin number and interval setting were varied to quantize SUVs within the MTV of cervical tumors. Subsequently, textural indices were calculated under different quantization methods and parameters. The final selection of the quantization method and parameter was determined by the performance in differentiating clinical endpoints. These approaches enabled us to use robust techniques for achieving better redundancy analysis and feature selection. Before translating our findings into the clinical practice, independent validation datasets are required to clarify the effects of different quantization methods and parameters to verify the clinical importance of texture analysis. In addition, there is a need for easy-to-use software tools for feature extraction and standardization for texture analysis^36^. By this way, more investigators can join the studies.

In a cervical cancer study, intratumoral heterogeneity based on MTV defined using a fixed threshold approach (a fixed threshold of 40% of the SUV_max_) was unable to predict outcomes in 73 patients receiving CRT^25^. The same investigators further explored additional metrics (sphericity, extent, and Shannon entropy) in another group of 85 patients with a FIGO stage of IIb and reported similar negative conclusions regarding the prediction of PLN involvement^26^. Regardless of previous negative studies, they reported the intriguing results of another study in which several textural metrics had a predictive value in the response to a therapy in 20 patients with cervical cancer when considering their temporal evolution from baseline to week 2, week 4, and post-therapy PET scans^12^. In particular, the figures showed a steady decline in HGZE, SZHGE, and LZHGE values in complete metabolic responders. However, this study did not correlate the prognostic impact of pretreatment textural index values with long-term treatment outcomes.

Our study disclosed that HGRE from GLRLM (the formula shown below) a predictor to discriminate several endpoints of treatment outcome in patients with cervical cancer.

$${\rm{HGRE}}=\frac{{\sum }_{i=1}^{{N}_{g}}{\sum }_{j=1}^{{N}_{r}}{i}^{2}\ast P(i,\,j|\theta )}{{\sum }_{i=1}^{{N}_{g}}{\sum }_{j=1}^{{N}_{r}}P(i,\,j|\theta )}$$

*P*(i, j|θ) is the entry (i, j) in the run-length matrix *P* for a direction θ, *N*_g_ is the number of bins used to discretize SUVs within MTV, and *N*_r_ is the number of different run lengths. HGRE measures the distribution of high SUVs within MTV and reflects a greater concentration of high SUVs by a high measurement. Therefore, the clinical impact of this feature indicates that the concentration of high SUVs might be an important factor in predicting CRT outcome. Interestingly, our previous study in patients with oropharyngeal or hypopharyngeal cancers uncovered that the HGRE values are associated with the expression of some protein biomarkers including *VEG*F, *GLUT-1*, *Claudin-4*, and *c-Met*^37^. Currently, the biological interpretation of the features on ^18^F-FDG-PET images of cervical cancer remains an area of our work.

Recently, Lucia *et al*. investigated a cohort of 102 cervical cancer patients and found that textural features such as gray-level nonuniformity for run (GLNUr) from GLRLM on ^18^F–FDG PET and Entropy (GLCM) derived from diffusion-weighted magnetic resonance imaging were independent prognostic factors for disease outcome^14^. In another study including 44 patients with bulky (≥4 cm) cervical cancer treated with CRT^13^, only one pretreatment factor, GLNUr (GLRLM), was associated with poor OS in univariate analysis. In their study, an interim PET-CT was performed 2 weeks after initiating CRT. The authors concluded that risk stratification by GLNUr and TLG decline showed similar prognostic prediction as post-CRT response, but much earlier. This provides an opportunity to adjust individualized regimens early in the treatment course. Similar results were not observed in our data; however, the area under ROC curves plotted by using GLNUr for calculating the events of OS, PFS, PRFS, and DMFS were 0.52 ± 0.06, 0.54 ± 0.05, 0.55 ± 0.06, and 0.53 ± 0.05, respectively. A plausible reason for this disparity may be the different approaches used to determine the quantization of MTV into a fixed number of bins or fixed intervals. The selection of the discretization affects the quantization results and transforms the performance of the features. Despite this difference, it should be emphasized that most studies have indicated that extracting more advanced image features from PET-CT can provide additional clinical information. Although the level of evidence is probably still insufficient, a positive trend can be observed.

None of the four groups of textural indices demonstrated predictive ability for distant metastasis in the separate cohorts, whole population or in patients with squamous cell carcinoma histology. The potential reason is that the textures of metastatic lymph nodes were not included in the analysis because of technical difficulty. For example, a spherical lesion with a diameter of 1 cm might contain only up to about 12 voxels (2 × 2 × 3) on PET images when the spacing and thickness are 5.47 (transaxial pixel width) and 3.27 (axial slice thickness) mm, respectively. Although performing textural analysis for MTV with a small number of voxels is challenging, a possible solution to circumvent this limitation is to improve the resolution of identification through an interpolation method, which is one of our ongoing investigations for advancing texture analysis.

Furthermore, several studies in lung cancer have investigated the potential combination of image-derived features from both PET and the low-dose CT component of the PET-CT dataset^38–40^. While PET images provide functional information about tumor uptake, CT images provide anatomical information and electron density measures that are useful for target definition and dose calculations. The main difference between the CT portion of PET-CT and diagnostic CT is the uses of radiation dose and contrast medium. The use of low radiation dose harms the image quality causing blurring effect in the measurement of heterogeneity^41^. Particularly when low-dose CT is applied in the area other than lung, the effect would be more prominent. Besides, the use of contrast medium enhances the visibility of internal structures and the surrounding tissues.

The findings of this study should be interpreted cautiously because they represent a retrospective study design in a single institute. External validation studies using an independent data set with imaging studies and a range of scanner manufacturers, resolution settings, and reconstruction algorithms are necessary to confirm these findings. Conducting such studies is crucial because textural features can be highly dependent on reconstruction schemes and imaging parameters^10,36^. Furthermore, texture analysis in patients with nonsquamous cell histology should be investigated further to maximize the prognostic value. In addition, although there is a need to correlate textural heterogeneity with any specific underlying biological heterogeneity, our findings provide a hint that future studies can clarify biological mechanisms that may be related to the value of HGRE^37^. Furthermore, the use of a fixed threshold at 50% of SUVmax to define MTV can potentially lead to underestimation of functional volumes especially in cases of high heterogeneity of intratumoral uptake^24^. Although this approach is generally based upon its simplicity and the ease of implementation, and could reduce the probability for the connection between the extracted MTV and the bladder, it will be imperative to compare our method with advanced segmentation algorithms using a contour-based or clustered-based approaches for different biological endpoints^36^. Finally, choosing the optimal number of bins for computing textural indices or determining the most robust features for a special clinical outcome should be guided by machine learning process. Nevertheless, the strengths of this study include the uniform treatment strategies, histological classification, and comprehensive analyses of textural indices. Our findings initiate a pivotal step to enable the tailoring of CRT to the specific textural features for patients with cervical cancer. After future validation studies, oncologists can rapidly assess the feasibility of salvage surgery, conduct dose escalation schemes, and initiate a novel combination therapy for high-risk patients.

## Conclusion

In patients with cervical cancer receiving definitive CRT, intratumoral heterogeneity employing the pretreatment textural features of HGRE on ^18^F-FDG PET-CT can supplement the prognostic information on OS, PFS, and local control along with the FIGO stage, and histological type. External validation studies with standard methods for texture analysis are warranted.

## Electronic supplementary material

Appendix files
