# Supplementary material for: Textural features of cervical cancers on FDG-PET/CT associate with survival and local relapse in patients treated with definitive chemoradiotherapy
Source: Sci Rep. 2018 Aug 8;8:11859. doi: 10.1038/s41598-018-30336-6 (PMC6082904; doi:10.1038/s41598-018-30336-6)
Supplement: Supplementary file 1 — Appendix files [file 41598_2018_30336_MOESM1_ESM.doc]

**Textural features of cervical cancers on FDG-PET/CT associate with survival and local relapse in patients treated with definitive chemoradiotherapy**

**Running title:** Textural features of cervical cancer on ^18^F-FDG PET associate with outcomes

Shang-Wen Chen ^1,2,3^, Wei-Chih Shen ^4,5^, Te-Chun Hsieh ^6,7^, Ji-An Liang ^1,8^, Yao-Ching Hung^2,9^, Lian-Shung Yeh ^2,9^, Wei-Chun Chang^2,9^, Wu-Chou Lin ^2,9^, Kuo-Yang Yen^6,7^, Chia-Hung Kao^6,8,10^

**Appendix 1**

Indices calculated from the textural analysis and the area under the receiver operating characteristic curve for predicting the lymph node status, FIGO stage, and histological type.

| **Classification of matrix** | **Index** |
| --- | --- |
| Conventional PET-related parameter | SUVmax |
|  | MTV |
|  | TLGmean |
| Gray Level Cooccurrence Matrix (GLCM) | Homogeneity |
|  | Energy |
|  | Correlation |
|  | Contrast |
|  | Entropy |
|  | Dissimilarity |
| Gray Level Run Length Matrix (GLRLM) | SRE |
|  | LRE |
|  | LGRE |
|  | HGRE |
|  | SRLGE |
|  | SRHGE |
|  | LRLGE |
|  | LRHGE |
|  | GLNUr |
|  | RLNU |
|  | RP |
| Neighborhood Gray-Level Different Matrix (NGLDM) | Coarseness |
|  | Contrast |
|  | Busyness |
| Gray Level Zone Length Matrix (GLZLM) | SZE |
|  | LZE |
|  | LGZE |
|  | HGZE |
|  | SZLGE |
|  | SZHGE |
|  | LZLGE |
|  | LZHGE |
|  | GLNUz |
|  | ZLNU |
|  | ZP |

Abbreviations: SRE = short-run emphasis; LRE = long-run emphasis; LGRE = low gray-level run emphasis; HGRE = high gray-level run emphasis; SRLGE = short-run low gray-level emphasis; SRHGE = short-run high gray-level emphasis; LRLGE = long-run low gray-level emphasis; LRHGE = long-run high gray-level emphasis; GLNUr = gray-level nonuniformity for run; RLNU = run-length nonuniformity; RP = run percentage; SZE = short-zone emphasis; LZE = long-zone emphasis; LGZE = low gray-level zone emphasis; HGZE = high gray-level zone emphasis; SZLGE= short-zone low gray-level emphasis; SZHGE = short-zone high gray-level emphasis; LZLGE = long-zone low gray-level emphasis; LZHGE = long-zone high gray-level emphasis; GLNUz = gray-level nonuniformity for zone; ZLNU = zone length nonuniformity; and ZP = zone percentage.

**Appendix 2**

**The performance of texture indices analyzed by ROC curve for comparing the effects of different discretization methods in terms of predicting pelvic recurrence. In each field, the significant Az was marked by gray.**

| Texture Index | |  |  | Discretization using a fixed number of bins | | | | | | | | | | | | | | | | | | | | | | | | | | | | | | | | | | | | | | |
| --- | --- | --- | --- | --- | --- | --- | --- | --- | --- | --- | --- | --- | --- | --- | --- | --- | --- | --- | --- | --- | --- | --- | --- | --- | --- | --- | --- | --- | --- | --- | --- | --- | --- | --- | --- | --- | --- | --- | --- | --- | --- | --- |
|  |  |  |  | 4 | | |  | 8 | | |  | 16 | | |  | 32 | | |  | 48 | | |  | 64 | | |  | 80 | | |  | 96 | | |  | 112 | | |  | 128 | | |
| GLCM | | |  | Az |  | Sig. |  | Az |  | Sig. |  | Az |  | Sig. |  | Az |  | Sig. |  | Az |  | Sig. |  | Az |  | Sig. |  | Az |  | Sig. |  | Az |  | Sig. |  | Az |  | Sig. |  | Az |  | Sig. |
|  | Homogeneity | |  | .532 |  | .634 |  | .542 |  | .535 |  | .558 |  | .385 |  | .559 |  | .384 |  | .567 |  | .32 |  | .55 |  | .46 |  | .546 |  | .49 |  | .541 |  | .537 |  | .531 |  | .648 |  | .541 |  | .543 |
|  | Energy | |  | .618 |  | .08 |  | .583 |  | .219 |  | .56 |  | .372 |  | .537 |  | .586 |  | .523 |  | .733 |  | .527 |  | .689 |  | .528 |  | .677 |  | .524 |  | .718 |  | .523 |  | .729 |  | .522 |  | .748 |
|  | Correlation | |  | .465 |  | .604 |  | .478 |  | .739 |  | .48 |  | .765 |  | .484 |  | .817 |  | .487 |  | .844 |  | .487 |  | .844 |  | .489 |  | .875 |  | .489 |  | .868 |  | .488 |  | .859 |  | .488 |  | .857 |
|  | Contrast | |  | .418 |  | .222 |  | .416 |  | .212 |  | .403 |  | .149 |  | .402 |  | .143 |  | .401 |  | .14 |  | .4 |  | .135 |  | .4 |  | .137 |  | .4 |  | .137 |  | .4 |  | .138 |  | .4 |  | .135 |
|  | Entropy | |  | .377 |  | .068 |  | .443 |  | .393 |  | .46 |  | .55 |  | .481 |  | .778 |  | .486 |  | .839 |  | .487 |  | .852 |  | .485 |  | .822 |  | .488 |  | .855 |  | .483 |  | .795 |  | .488 |  | .857 |
|  | Dissimilarity | |  | .441 |  | .379 |  | .421 |  | .241 |  | .408 |  | .172 |  | .401 |  | .14 |  | .401 |  | .141 |  | .399 |  | .132 |  | .4 |  | .137 |  | .399 |  | .132 |  | .4 |  | .137 |  | .398 |  | .131 |
| GLRLM | | |  |  |  |  |  |  |  |  |  |  |  |  |  |  |  |  |  |  |  |  |  |  |  |  |  |  |  |  |  |  |  |  |  |  |  |  |  |  |  |  |
|  | SRE | |  | .505 |  | .942 |  | .52 |  | .769 |  | .473 |  | .685 |  | .458 |  | .531 |  | .464 |  | .59 |  | .497 |  | .96 |  | .528 |  | .677 |  | .517 |  | .8 |  | .532 |  | .636 |  | .516 |  | .813 |
|  | LRE | |  | .502 |  | .975 |  | .47 |  | .652 |  | .48 |  | .765 |  | .495 |  | .944 |  | .534 |  | .61 |  | .485 |  | .826 |  | .469 |  | .646 |  | .437 |  | .345 |  | .439 |  | .364 |  | .491 |  | .899 |
|  | LGRE | |  | .65 |  | .025 |  | .634 |  | .046 |  | .621 |  | .072 |  | .587 |  | .195 |  | .565 |  | .332 |  | .582 |  | .221 |  | .534 |  | .616 |  | .526 |  | .701 |  | .544 |  | .511 |  | .495 |  | .946 |
|  | HGRE | |  | .313 |  | .005 |  | .322 |  | .008 |  | .318 |  | .007 |  | .321 |  | .008 |  | .323 |  | .008 |  | .322 |  | .008 |  | .325 |  | .009 |  | .323 |  | .008 |  | .323 |  | .009 |  | .325 |  | .009 |
|  | SRLGE | |  | .666 |  | .013 |  | .644 |  | .033 |  | .622 |  | .069 |  | .582 |  | .221 |  | .568 |  | .312 |  | .587 |  | .194 |  | .533 |  | .624 |  | .526 |  | .701 |  | .545 |  | .508 |  | .496 |  | .951 |
|  | SRHGE | |  | .346 |  | .022 |  | .329 |  | .011 |  | .309 |  | .005 |  | .315 |  | .006 |  | .316 |  | .006 |  | .319 |  | .007 |  | .325 |  | .009 |  | .324 |  | .009 |  | .322 |  | .008 |  | .325 |  | .009 |
|  | LRLGE | |  | .595 |  | .155 |  | .621 |  | .073 |  | .603 |  | .127 |  | .585 |  | .208 |  | .559 |  | .376 |  | .584 |  | .21 |  | .542 |  | .531 |  | .521 |  | .75 |  | .545 |  | .506 |  | .496 |  | .955 |
|  | LRHGE | |  | .365 |  | .044 |  | .315 |  | .006 |  | .33 |  | .011 |  | .334 |  | .014 |  | .325 |  | .009 |  | .325 |  | .009 |  | .323 |  | .008 |  | .325 |  | .009 |  | .319 |  | .007 |  | .322 |  | .008 |
|  | GLNUr | |  | .552 |  | .443 |  | .55 |  | .46 |  | .545 |  | .506 |  | .541 |  | .543 |  | .537 |  | .579 |  | .54 |  | .556 |  | .541 |  | .544 |  | .542 |  | .533 |  | .543 |  | .526 |  | .545 |  | .5 |
|  | RLNU | |  | .525 |  | .71 |  | .526 |  | .704 |  | .512 |  | .861 |  | .506 |  | .93 |  | .504 |  | .948 |  | .504 |  | .955 |  | .505 |  | .935 |  | .507 |  | .917 |  | .503 |  | .964 |  | .502 |  | .973 |
|  | RP | |  | .537 |  | .577 |  | .541 |  | .544 |  | .502 |  | .971 |  | .516 |  | .808 |  | .506 |  | .926 |  | .502 |  | .982 |  | .512 |  | .857 |  | .507 |  | .921 |  | .505 |  | .937 |  | .512 |  | .859 |
| NGLDM | | |  |  |  |  |  |  |  |  |  |  |  |  |  |  |  |  |  |  |  |  |  |  |  |  |  |  |  |  |  |  |  |  |  |  |  |  |  |  |  |  |
|  | Coarseness | |  | .468 |  | .634 |  | .457 |  | .524 |  | .459 |  | .543 |  | .47 |  | .654 |  | .473 |  | .685 |  | .472 |  | .681 |  | .473 |  | .687 |  | .47 |  | .66 |  | .473 |  | .683 |  | .482 |  | .787 |
|  | Contrast | |  | .437 |  | .349 |  | .459 |  | .541 |  | .471 |  | .662 |  | .476 |  | .716 |  | .48 |  | .763 |  | .482 |  | .793 |  | .488 |  | .859 |  | .485 |  | .826 |  | .487 |  | .85 |  | .484 |  | .808 |
|  | Busyness | |  | .58 |  | .232 |  | .583 |  | .215 |  | .587 |  | .193 |  | .584 |  | .211 |  | .585 |  | .205 |  | .588 |  | .189 |  | .589 |  | .187 |  | .59 |  | .179 |  | .593 |  | .165 |  | .594 |  | .16 |
| GLZLM | | |  |  |  |  |  |  |  |  |  |  |  |  |  |  |  |  |  |  |  |  |  |  |  |  |  |  |  |  |  |  |  |  |  |  |  |  |  |  |  |  |
|  | SZE | |  | .589 |  | .185 |  | .442 |  | .385 |  | .478 |  | .748 |  | .509 |  | .897 |  | .513 |  | .85 |  | .565 |  | .334 |  | .526 |  | .701 |  | .531 |  | .644 |  | .522 |  | .746 |  | .52 |  | .769 |
|  | LZE | |  | .519 |  | .782 |  | .511 |  | .875 |  | .509 |  | .892 |  | .448 |  | .443 |  | .457 |  | .522 |  | .436 |  | .342 |  | .442 |  | .388 |  | .449 |  | .45 |  | .469 |  | .64 |  | .486 |  | .83 |
|  | LGZE | |  | .403 |  | .149 |  | .51 |  | .879 |  | .603 |  | .125 |  | .625 |  | .064 |  | .606 |  | .113 |  | .586 |  | .199 |  | .55 |  | .453 |  | .553 |  | .43 |  | .537 |  | .581 |  | .539 |  | .558 |
|  | HGZE | |  | .549 |  | .469 |  | .424 |  | .256 |  | .311 |  | .005 |  | .306 |  | .004 |  | .32 |  | .008 |  | .318 |  | .007 |  | .315 |  | .006 |  | .317 |  | .006 |  | .317 |  | .007 |  | .327 |  | .01 |
|  | SZLGE | |  | .503 |  | .964 |  | .425 |  | .264 |  | .523 |  | .731 |  | .639 |  | .039 |  | .618 |  | .08 |  | .598 |  | .144 |  | .558 |  | .391 |  | .572 |  | .284 |  | .54 |  | .55 |  | .556 |  | .401 |
|  | SZHGE | |  | .626 |  | .06 |  | .475 |  | .71 |  | .353 |  | .029 |  | .329 |  | .011 |  | .333 |  | .013 |  | .327 |  | .01 |  | .314 |  | .006 |  | .316 |  | .006 |  | .324 |  | .009 |  | .336 |  | .015 |
|  | LZLGE | |  | .556 |  | .404 |  | .598 |  | .146 |  | .594 |  | .16 |  | .526 |  | .701 |  | .527 |  | .689 |  | .528 |  | .672 |  | .509 |  | .892 |  | .515 |  | .822 |  | .516 |  | .817 |  | .477 |  | .727 |
|  | LZHGE | |  | .474 |  | .697 |  | .425 |  | .262 |  | .387 |  | .093 |  | .319 |  | .007 |  | .308 |  | .004 |  | .299 |  | .003 |  | .317 |  | .006 |  | .305 |  | .004 |  | .311 |  | .005 |  | .31 |  | .005 |
|  | GLNUz | |  | .564 |  | .344 |  | .543 |  | .524 |  | .562 |  | .355 |  | .561 |  | .364 |  | .55 |  | .453 |  | .548 |  | .479 |  | .543 |  | .519 |  | .543 |  | .52 |  | .544 |  | .513 |  | .547 |  | .488 |
|  | ZLNU | |  | .591 |  | .178 |  | .509 |  | .899 |  | .51 |  | .883 |  | .538 |  | .573 |  | .523 |  | .729 |  | .531 |  | .646 |  | .518 |  | .785 |  | .519 |  | .78 |  | .508 |  | .901 |  | .512 |  | .855 |
|  | ZP | |  | .45 |  | .453 |  | .469 |  | .648 |  | .566 |  | .329 |  | .593 |  | .165 |  | .616 |  | .083 |  | .546 |  | .49 |  | .527 |  | .685 |  | .531 |  | .644 |  | .514 |  | .835 |  | .52 |  | .763 |

| Texture Index | |  | Discretization using fixed bin width | | | | | | | | | | | | | | | | | | | | | | | | | | | | | | | | | | |
| --- | --- | --- | --- | --- | --- | --- | --- | --- | --- | --- | --- | --- | --- | --- | --- | --- | --- | --- | --- | --- | --- | --- | --- | --- | --- | --- | --- | --- | --- | --- | --- | --- | --- | --- | --- | --- | --- |
|  |  |  | .01 | | |  | .025 | | |  | .05 | | |  | .075 | | |  | .1 | | |  | .25 | | |  | .5 | | |  | .75 | | |  | 1 | | |
| GLCM | | | Az |  | Sig. |  | Az |  | Sig. |  | Az |  | Sig. |  | Az |  | Sig. |  | Az |  | Sig. |  | Az |  | Sig. |  | Az |  | Sig. |  | Az |  | Sig. |  | Az |  | Sig. |
|  | Homogeneity | | .545 |  | .499 |  | .555 |  | .414 |  | .56 |  | .37 |  | .563 |  | .352 |  | .559 |  | .382 |  | .563 |  | .346 |  | .55 |  | .457 |  | .564 |  | .344 |  | .54 |  | .554 |
|  | Energy | | .511 |  | .872 |  | .527 |  | .689 |  | .545 |  | .502 |  | .552 |  | .443 |  | .56 |  | .373 |  | .569 |  | .305 |  | .565 |  | .335 |  | .568 |  | .31 |  | .572 |  | .287 |
|  | Correlation | | .491 |  | .888 |  | .489 |  | .87 |  | .489 |  | .875 |  | .491 |  | .888 |  | .489 |  | .866 |  | .486 |  | .839 |  | .475 |  | .714 |  | .481 |  | .774 |  | .458 |  | .535 |
|  | Contrast | | .432 |  | .31 |  | .432 |  | .31 |  | .432 |  | .31 |  | .432 |  | .31 |  | .432 |  | .31 |  | .431 |  | .305 |  | .431 |  | .302 |  | .429 |  | .292 |  | .436 |  | .341 |
|  | Entropy | | .496 |  | .957 |  | .48 |  | .761 |  | .469 |  | .644 |  | .469 |  | .648 |  | .463 |  | .577 |  | .445 |  | .417 |  | .441 |  | .376 |  | .432 |  | .313 |  | .43 |  | .3 |
|  | Dissimilarity | | .433 |  | .316 |  | .432 |  | .31 |  | .432 |  | .312 |  | .431 |  | .308 |  | .433 |  | .316 |  | .434 |  | .324 |  | .436 |  | .344 |  | .431 |  | .302 |  | .443 |  | .398 |
| GLRLM | | |  |  |  |  |  |  |  |  |  |  |  |  |  |  |  |  |  |  |  |  |  |  |  |  |  |  |  |  |  |  |  |  |  |  |  |
|  | SRE | | .521 |  | .75 |  | .454 |  | .495 |  | .48 |  | .767 |  | .448 |  | .442 |  | .44 |  | .37 |  | .412 |  | .191 |  | .458 |  | .531 |  | .441 |  | .379 |  | .476 |  | .72 |
|  | LRE | | .479 |  | .75 |  | .545 |  | .502 |  | .52 |  | .763 |  | .554 |  | .422 |  | .547 |  | .486 |  | .572 |  | .282 |  | .529 |  | .664 |  | .558 |  | .388 |  | .522 |  | .742 |
|  | LGRE | | .541 |  | .543 |  | .55 |  | .457 |  | .553 |  | .433 |  | .581 |  | .225 |  | .561 |  | .361 |  | .589 |  | .183 |  | .601 |  | .134 |  | .603 |  | .124 |  | .598 |  | .144 |
|  | HGRE | | .419 |  | .225 |  | .419 |  | .23 |  | .419 |  | .23 |  | .418 |  | .221 |  | .419 |  | .228 |  | .417 |  | .219 |  | .417 |  | .215 |  | .418 |  | .221 |  | .418 |  | .221 |
|  | SRLGE | | .541 |  | .543 |  | .55 |  | .457 |  | .553 |  | .43 |  | .582 |  | .221 |  | .561 |  | .364 |  | .588 |  | .191 |  | .603 |  | .125 |  | .611 |  | .099 |  | .602 |  | .131 |
|  | SRHGE | | .419 |  | .225 |  | .419 |  | .228 |  | .419 |  | .228 |  | .419 |  | .225 |  | .419 |  | .225 |  | .42 |  | .236 |  | .419 |  | .23 |  | .427 |  | .277 |  | .43 |  | .3 |
|  | LRLGE | | .541 |  | .539 |  | .551 |  | .45 |  | .553 |  | .43 |  | .583 |  | .217 |  | .57 |  | .294 |  | .587 |  | .195 |  | .587 |  | .197 |  | .594 |  | .162 |  | .561 |  | .361 |
|  | LRHGE | | .418 |  | .223 |  | .419 |  | .23 |  | .418 |  | .223 |  | .42 |  | .232 |  | .417 |  | .217 |  | .414 |  | .199 |  | .401 |  | .141 |  | .411 |  | .183 |  | .393 |  | .111 |
|  | GLNUr | | .566 |  | .325 |  | .57 |  | .297 |  | .571 |  | .289 |  | .571 |  | .289 |  | .567 |  | .321 |  | .564 |  | .341 |  | .567 |  | .318 |  | .561 |  | .364 |  | .561 |  | .364 |
|  | RLNU | | .504 |  | .953 |  | .502 |  | .975 |  | .502 |  | .975 |  | .503 |  | .964 |  | .503 |  | .962 |  | .5 |  | .996 |  | .501 |  | .989 |  | .488 |  | .855 |  | .495 |  | .946 |
|  | RP | | .512 |  | .852 |  | .512 |  | .852 |  | .523 |  | .731 |  | .522 |  | .739 |  | .54 |  | .548 |  | .523 |  | .729 |  | .498 |  | .973 |  | .486 |  | .83 |  | .514 |  | .833 |
| NGLDM | | |  |  |  |  |  |  |  |  |  |  |  |  |  |  |  |  |  |  |  |  |  |  |  |  |  |  |  |  |  |  |  |  |  |  |  |
|  | Coarseness | | .534 |  | .612 |  | .509 |  | .888 |  | .496 |  | .951 |  | .476 |  | .718 |  | .468 |  | .632 |  | .469 |  | .64 |  | .47 |  | .66 |  | .472 |  | .681 |  | .486 |  | .83 |
|  | Contrast | | .455 |  | .502 |  | .463 |  | .584 |  | .464 |  | .588 |  | .46 |  | .554 |  | .459 |  | .539 |  | .436 |  | .341 |  | .422 |  | .245 |  | .406 |  | .16 |  | .406 |  | .164 |
|  | Busyness | | .569 |  | .308 |  | .577 |  | .255 |  | .579 |  | .241 |  | .58 |  | .234 |  | .581 |  | .228 |  | .581 |  | .228 |  | .582 |  | .223 |  | .58 |  | .234 |  | .58 |  | .236 |
| GLZLM | | |  |  |  |  |  |  |  |  |  |  |  |  |  |  |  |  |  |  |  |  |  |  |  |  |  |  |  |  |  |  |  |  |  |  |  |
|  | SZE | | .545 |  | .506 |  | .467 |  | .62 |  | .474 |  | .701 |  | .466 |  | .612 |  | .478 |  | .748 |  | .502 |  | .978 |  | .484 |  | .817 |  | .541 |  | .539 |  | .488 |  | .857 |
|  | LZE | | .449 |  | .447 |  | .53 |  | .656 |  | .528 |  | .672 |  | .524 |  | .722 |  | .518 |  | .791 |  | .525 |  | .71 |  | .536 |  | .588 |  | .523 |  | .727 |  | .541 |  | .543 |
|  | LGZE | | .542 |  | .535 |  | .555 |  | .417 |  | .548 |  | .471 |  | .58 |  | .236 |  | .57 |  | .3 |  | .579 |  | .239 |  | .537 |  | .584 |  | .539 |  | .558 |  | .487 |  | .846 |
|  | HGZE | | .419 |  | .23 |  | .42 |  | .236 |  | .422 |  | .245 |  | .424 |  | .257 |  | .424 |  | .259 |  | .433 |  | .316 |  | .446 |  | .42 |  | .447 |  | .433 |  | .453 |  | .486 |
|  | SZLGE | | .546 |  | .492 |  | .557 |  | .395 |  | .55 |  | .457 |  | .584 |  | .213 |  | .572 |  | .287 |  | .613 |  | .093 |  | .552 |  | .437 |  | .523 |  | .735 |  | .501 |  | .987 |
|  | SZHGE | | .421 |  | .241 |  | .423 |  | .25 |  | .427 |  | .277 |  | .431 |  | .305 |  | .431 |  | .305 |  | .446 |  | .423 |  | .461 |  | .565 |  | .506 |  | .928 |  | .481 |  | .774 |
|  | LZLGE | | .535 |  | .6 |  | .541 |  | .539 |  | .544 |  | .513 |  | .555 |  | .414 |  | .544 |  | .509 |  | .555 |  | .41 |  | .553 |  | .43 |  | .55 |  | .453 |  | .559 |  | .379 |
|  | LZHGE | | .413 |  | .197 |  | .416 |  | .211 |  | .406 |  | .162 |  | .4 |  | .135 |  | .389 |  | .1 |  | .375 |  | .062 |  | .463 |  | .584 |  | .473 |  | .693 |  | .494 |  | .933 |
|  | GLNUz | | .57 |  | .294 |  | .573 |  | .278 |  | .573 |  | .279 |  | .565 |  | .332 |  | .571 |  | .289 |  | .528 |  | .672 |  | .508 |  | .908 |  | .516 |  | .813 |  | .518 |  | .789 |
|  | ZLNU | | .506 |  | .93 |  | .497 |  | .962 |  | .501 |  | .993 |  | .498 |  | .98 |  | .499 |  | .984 |  | .489 |  | .875 |  | .476 |  | .722 |  | .491 |  | .897 |  | .469 |  | .642 |
|  | ZP | | .54 |  | .554 |  | .516 |  | .808 |  | .531 |  | .644 |  | .505 |  | .939 |  | .542 |  | .535 |  | .476 |  | .72 |  | .478 |  | .746 |  | .493 |  | .915 |  | .465 |  | .606 |

Abbreviations: as Appendix 1. Az: the area under the ROC curve.

**Appendix 3**

Patients with squamous cell carcinoma (n = 114).

Figure A1a. Overall survival according to tumors with a high and low HGRE value (P = .001).

Overall survival (years)

Figure A1b. Pelvic relapse-free survival according to tumors with a high and low HGRE value (P = .006).

Pelvic relapse-free survival (years)

**Appendix 4**

Subgroup analysis in patients with FIGO stage IB-IIB and FIGO IIIA-IVA diseases.

Patients with FIGO stage IB-IIB (n = 115)

Figure A2a.Overall survival according to tumors with a high and low HGRE value (P = .001).

Overall survival (years)

Figure A2b. Pelvic relapse-free survival according to tumors with a high and low HGRE value (P < .0001).

Pelvic relapse-free survival (years)
